# Probiotic evaluation, adherence capability and safety assessment of *Lactococcus lactis* strain isolated from an important herb “*Murraya koenigii*”

**DOI:** 10.1038/s41598-024-66597-7

**Published:** 2024-07-06

**Authors:** Tholla Madana Shivani, Mythili Sathiavelu

**Affiliations:** School of Biosciences and Technology, Vellore Institute of Technology, Vellore, 632014 India

**Keywords:** *Murraya koenigii*, Adhesion ability, Hydrophobicity, *Lactococcus lactis*, Antagonistic activity, Biotechnology, Microbiology

## Abstract

Lactic acid bacteria (LAB) isolated from medicinal herb *Murraya koenigii*, commonly known as curry leaf, which promotes the growth and maintenance of gut microbiota, were studied for their probiotic potential. The key objective of this research was to isolate and evaluate probiotic characteristics, test adherence capabilities, and confirm their safety. *Lactococcus lactis* (MKL8), isolated from *Murraya koenigii,* was subjected to in vitro analysis to assess its resistance to the gastric environment, ability to adhere Caco-2 cells, anti-microbial activity, hydrophobicity, auto-aggregation, and safety profiling through MTT assay and hemolytic. MKL8 exhibited growth at 0.5% phenol concentrations (> 80%) and was able to survive in conditions with high bile concentrations (> 79%) and a relatively low pH (72%-91%). It shows high tolerance to high osmotic conditions (> 73%) and simulated gastric juice (> 72%). Additionally, MKL8 demonstrated strong hydrophobicity (85%), auto-aggregation (87.3%-91.7%), and adherence to Caco-2 cells. Moreover, it had an inhibitory effect against pathogens too. By performing the hemolytic and MTT assays, the non-toxicity of MKL8 isolate was examined, and it exhibited no harmful characteristics. Considering MKL8's resistance to gastrointestinal tract conditions, high surface hydrophobicity, non-toxicity, and ability to inhibit the tested pathogens, it can be concluded that MKL8 demonstrated promising probiotic properties and has potential for use in the food industry.

## Introduction

Many plants harbour lactic acid bacteria (LAB) in their endosphere, phyllosphere, and rhizosphere. The ability of LAB to survive and thrive in the plant endosphere implies a close relationship that is responsible for raising crop yield by increasing food availability, serving as a biocontrol agent, and minimizing abiotic and biotic stress^1^. The expanding usage of dairy products in the food industry led this study to explore whether endosphere bacteria may be used as a potential source for isolating probiotic bacteria. Isolating probiotic bacteria from novel, economical, non-traditional sources that don’t have detrimental impact on health is becoming more and more important. Therefore, plant leaves have become be prospective source for isolation of bacterial genera such as *Lactococcus, Lactobacillus, Enterococcus, Enterobacter, Pediococcus* and, *Staphylococcus*^2,3^. Of all these, *Lactococcus* is the most widely distributed in many habitats. It is frequently found among various parts of different plants, making it a prominent endophyte. Studies were previously reported wherein *Lactococcus lactis* was isolated from eucalyptus stems^4^, aquatic plants^5^, from internal tissues and leaves of sugarcane^6^, fresh *Pisum sativum* and baby corn,^7^ and from *Lippia sidoides Cham*. (pepper-rosmarin)^8^. However, the functional importance of numerous groups of microbes, in particular the lactic acid bacteria that inhabit *Murraya koenigii*, still needs further exploration.

*Murraya koenigii* is indigenous to India, Sri Lanka, and several South Asian countries. It is a leafy vegetable in the Rutaceae family commonly called curry leaf. Fresh *Murraya koenigii* leaves contain volatile oil as part of their chemical composition. This herb's stems, leaves, and roots contain carbazole alkaloids and triterpenes^9^. Due to the diversity of flavonoids, carboxylic alkaloids, and dietary phenols, *Murraya koenigii* has been renowned for its wide spectrum of therapeutic properties.

Medicinal plant species also have unique microbiomes within the plant kingdom because of the presence of their distinctive and structurally diverse secondary metabolites. Since the discovery of the plant-associated endophytic microbiome, interactions between medicinal plants and bacteria have caught the attention of researchers globally^10^. Given the many therapeutic benefits of *M. koenigii*, such as its ability to combat diabetes and cancer, the discovery of possible probiotic endophytes in the leaves of the plant would be an advantageous addition that could be used to prepare probiotics for commercial purposes. Consequently, the goal of the study was to isolate and identify potential probiotic endophytic bacteria from *Murraya koenigii*. All the isolated cultures were tested for probiotic characteristics, primarily acid (low pH), bile salt, osmotic and, phenol resistance. The positive strain with probiotic potential was further evaluated using a variety of parameters, including antibacterial activity against food pathogens, in vitro adhesion capacity, and growth responses under various stress conditions.

## Materials and methods

### Acquiring media and cell-line

All of the media utilized in the present study were acquired from Hi-Media and Sisco Research Laboratories Pvt. Ltd. (SRL)—India. The Caco-2 cell lines were acquired from the National Centre for Cell Science in Pune, India.

### Sample collection, isolation, and phenotypic characterization of endophytic LAB

Healthy and mature *Murraya koenigii* plants were carefully selected and collected from the garden in Vellore, Tamil Nadu, and brought to the laboratory in sterile zip-lock bag. Initially, the explants were cleansed under running tap water. To gently reduce surface tension and enable the sterilizing agent to get into niches and grooves beyond the epidermal cells, leaf sample was surface sterilized using 70% ethanol for 5 min, washed with sterile water, and then treated with 7–10 drops of Tween 20 for 10 min. It was subsequently followed by immersing the leaf sample in 0.1% Mercuric chloride for 5 min, and the explants were washed three times with sterilised distilled water in order to get rid of the disinfectants^11^. The sterilized leaf was then used to isolate the endophytic LAB by macerating using mortar and pestle followed by platin over selective solid MRS media (De Man, Rogosa, and Sharpe) (Hi-media GM369-500 g) and incubated at 28 °C; 2 days to maximise the recovery of the entophytes.

The examination of sterility for sterile explants was confirmed in two ways:

1. Surface-sterilized leaves were imprinted into MRS media.
2. The final rinse water was plated over MRS media

Surface sterilization is deemed successful if the control petri-plate shows no signs of microbial development. Obtained bacteria were tested for antimicrobial activity against pathogens (section "Agar well diffusion assay"). To obtain pure endophytic isolates, the bacterial cultures were repeatedly streaked on the MRS agar plates, and isolates were stored at 4 °C using glycerol stock. The pure isolate was preliminary identified by standard biochemical assays, motility, gram staining, and a catalase test using 3% v/v H_2_O_2_.

### Characteristics of bacteria associated with probiotic activity

#### Resistance to acid and bile salts

Using the methodology developed by Prabhurajeshwar, and Chandrakanth, the growth potential of the strain in the presence of bile (w/v) and at different pH levels was assessed with slight modifications. After overnight culturing, 1 ml of the bacterial culture was added into MRS broth containing sterilized bile (ox-gall) (SRL-99455) at concentrations of 0.2%, 0.4%, 0.6%, 0.8%, and 1%. Additionally, MRS broth with different pH levels ranging from 2 and 10 was prepared. Following a 4-h incubation period at 37 °C, absorbance at 600 nm was measured using a UV–Vis spectrophotometer (SHIMADZU UV-1280) of the test and compared with a control culture that had a neutral pH and no bile^12^. Results were expressed as a growth percentage compared to control group and calculated using the given formula. Triplicate assays were performed.

$$\% {\text{ of survivability }} = {\text{ A}}_{{\text{o}}} - {\text{M}}_{{\text{o}}} /{\text{A}}_{{\text{o}}} \times {1}00$$

wherein A_o_ = growth of control; M_o_ = growth of test

$$\% {\text{ of survivability }} = {\text{ A}}_{{\text{o}}} - {\text{M}}_{{\text{o}}} /{\text{A}}_{{\text{o}}} \times {1}00$$

wherein A_o_ = growth of control; M_o_ = growth of test.

#### Resistance to phenol

The potential of LAB to tolerate phenol was assessed by culturing it in MRS broth with a increasing concentrations of phenol (SRL-28104) from (0.1–0.4%). 1% (v/v) overnight bacteria test culture was added into each tube consisting MRS broth with a specific phenol content after sterilization, and were then incubated at 37 °C for 24 h. Absorbance at a wavelength of 600 nm was measured after incubation to evaluate the viability of the strains. Findings were expressed as a growth percentage compared to the control group (unmodified) and calculated using the given formula. The test was conducted three times^13^.

$$\% {\text{ of survivability }} = {\text{ A}}_{{\text{o}}} - {\text{M}}_{{\text{o}}} /{\text{A}}_{{\text{o}}} \times {1}00$$

wherein A_o_ = growth of control; M_o_ = growth of test.

#### Resistance to NaCl

MRS broth with varying concentrations of NaCl, Hi-media Sodium Chloride, Hi-LR™ (GRM031) ranging from (0, 2, 4, 6, 8, and 10%) was inoculated with overnight incubated bacterial culture (1% v/v) and incubated for 24 h at 37 °C. The viability of the strains was evaluated by measure of absorbance at a wavelength of 600 nm, and was correlated with the control with no modifications. The result was represented in growth percentage, and the experiment was conducted in triplicate^14^.

$$\% {\text{ of survivability }} = {\text{ A}}_{{\text{o}}} - {\text{M}}_{{\text{o}}} /{\text{A}}_{{\text{o}}} \times {1}00$$

wherein A_o_ = growth of control; M_o_ = growth of test.

#### Resistance to simulated gastric juice

Bacteria were harvested from 24-h cultures (6000×*g*, 15 min, 4 °C), washed twice in phosphate-buffered saline (PBS) (0.02 M) (pH 6.8), and then suspended in Simulated Gastric Juice (SGJ), a solution of pH 3 with PBS mixed with pepsin (0.3 mg/mL, HiMedia), 45 mM NaHCO_3_ (SRL-56398), and 7 mM KCl (SRL-38630). The inoculated SGJ was incubated at 120 rpm on an orbital shaker to simulate peristalsis. The samples were collected at various time interval of 30, 60, 90, and 120 min and checked for absorbance at 600 nm. Control was measured subsequently with only PBS and bacteria without SGJ, and the survivable percentage was calculated using the given formula and the experiment was conducted in triplicate. After that, 0.1 ml of sample from each time was seeded on MRS agar plates to ensure viability and incubated for period of 24 h at 37 °C^15^.

$$\% {\text{ of survivability }} = {\text{ A}}_{{\text{o}}} - {\text{M}}_{{\text{o}}} /{\text{A}}_{{\text{o}}} \times {1}00$$

wherein A_o_ = growth of control; M_o_ = growth of test.

### Cell surface and adherence characteristics

#### Autoaggregation assay

Aggregates formed during cell sedimentation serve as a visual representation of auto aggregation. In order to assess the auto-aggregation, the bacterial cells were cultured to concentration 1 × 10^8^ CFU/ml and harvested (6000×*g*, 10 min). The pellet was washed with sterile PBS, suspended, and vortexed for 20 s. At intervals of 0, 2, 4, 6, 8, and 10 h after allowing the sample to sit at room temperature, absorbance at a wavelength of 600 nm of the uppermost suspension was assessed^16^. The experiment was carried out in triplicates.

The percentage of auto aggregation was calculated using the below formula:

$$\% {\text{ of Auto aggregation }} = \, \left[ {{1} - \left( {{\text{M}}_{{{\text{time}}}} /{\text{M}}_{0} } \right){ 1}00} \right]$$

M_time_ = absorbance at different time intervals; M_0_ = absorbance at 0 h.

#### Cell surface hydrophobicity

The Bacterial Adhesion to Hydrocarbons (BATH) experiment was used to determine the Cell Surface Hydrophobicity (CSH) of bacteria. Overnight bacterial culture (1 × 10^8^ CFU/ml) pellet was collected by centrifuging for 8000×*g*; 10 min. The pellet was rinsed with PBS buffer, re-suspended, and adjusted to an OD of 1.0 ± 0.05. In a glass test tube, 3 ml cell suspension was added, followed by 1 ml of hydrocarbon (n-hexane) (SRL-65764). The tubes were vortexed for 15–20 s after incubation of 10 min at 37 °C to achieve a steady temperature. Phase separation was achieved by maintaining the cell suspensions undisturbed at 37 °C for 20 min, and the absorbance at 600 nm was measured using lower aqueous phase^17^.

The percentage of hydrophobicity was calculated by

$$\% {\text{ Hydrophobicity}} = \left( {{\text{ODi}}{-}{\text{ODt}}/{\text{ODi}}} \right) \times {1}00;$$

wherein, ODi stands for the initial OD600 of cell suspension, and ODt stands for OD600 of aqueous recorded at time t.

#### In vitro cell adhesion assay using Caco-2 cells

##### Preparing cell lines and LAB

Using 12-well plates (Tarson, cat:980040), human colon adenocarcinoma cell line (Caco-2) was plated at concentration of 1 × 10^4^ cells/well in MEM medium with 1X antibiotic antimycotic solution (Code no: A002A-100ML, Hi-media) + 10% foetal bovine serum and incubated in CO_2_ incubator (5% CO_2_) at 37° C. Once every two days, the cultural medium was changed. After 15 days of post confluence, the cells were used after they have reached complete differentiation. 200 μl ml of PBS (pH 7.4) was used to wash the Caco-2 cells twice in monolayer. Before inoculating the bacteria, 1 mL of MEM devoid of antibiotics and serum was added to every well, followed by incubating it for 2 h at 37 °C. Bacterial culture (approximately 1 × 10^4^ CFU), which was suspended in 1 ml of MEM without serum, and antibiotics were then inoculated in each individual well of tissue culture plates with pre-adhered Caco-2 cell line. The plates were incubated in 5% CO_2_, 95% air for 2 h at 37 °C^18,19^.

##### Cell adherence and percent adhesion assay

After being incubated, non-adherent bacteria were eliminated from the monolayer by washing it five times in PBS (pH 7.4). The bacteria adhered were fixed using 1 ml of methanol and incubated for ten minutes at room temperature prior examination under a microscope. Following the methanol removal process, Giemsa stain solution i.e., 1:20 dilution-Giemsa in PBS was added to the cells to stain them. After incubating for 20 min at room temperature, plates were thoroughly washed with distilled water. The plates were allowed to air-dry under laminar air-flow and observed using an OPTIKA Inverted microscope (IM3FL4, ITALY). To determine percent adhesion, cells from the monolayer were separated by trypsinization for measuring the live adherent bacteria. 1 ml of 0.25% Trypsin–EDTA solution was added to each well followed by a 15-min incubation at room temperature. The lysed cell and bacterial suspension were plated on MRS agar after serial dilution with saline solution, and enumeration was performed after 24 h of incubation at 37 °C in an anaerobic atmosphere^18,19^.

Adhesion was quantified as a % of bacteria adhered relative to the total bacteria used in the experiment and measured as: % adhesion = (M_1_/M_0_) × 100, where M_0_ and M_1_ CFU/mL are the initial and final count of bacteria. The complete assay was performed in triplicates.

### Antagonistic activity of bacteria

#### Agar well diffusion assay

The agar well diffusion method was utilized to ascertain the antagonistic impact of the LAB against certain pathogens. The targeted pathogens and the LAB isolates were pre-cultured in Brain Heart Infusion (BHI) broth (Hi-media M2101-500 g) and MRS broth (Hi-media GM369-500 g), respectively, under the same conditions and incubated overnight temperature at 37 °C. Precisely 200 μl of test pathogens, containing 10^7^ CFU/ml, was applied over the surface of Mueller–Hinton Agar plates and swabbed. Then, 100 μl of cell-free supernatant (CFS) harvested by centrifuging (REMI C-24BL) for 6000×*g* for 10 min was added into wells punctured on the plates inoculated with the test pathogen. Plates were then incubated at 37 °C for 24 h. To evaluate LAB strains' antagonistic activity, inhibition zone (mm) surrounding wells were measured. The three common food and intestinal pathogens considered in the study were *Bacillus cereus* (MTCC 8776), *Klebsiella pneumoniae* (MTCC 2653), and *Escherichia coli* (MTCC 1564)^20^.

### Safety evaluation of LAB

#### Hemolytic assay

To assess the hemolytic activity of LAB isolates, the Kondrotiene et al.^21^ technique was employed. Blood agar (Hi-media M073-500 g) plates consisting of 5% (v/v) sheep blood was streaked with LAB (1 × 10^8^ CFU/ml) cultured in MRS broth, and plates were subsequently incubated for 24 h at 37 °C. The presence of the following showed the strains' hemolytic activities: Alpha (α) hemolysis, which is shown by a small area of the media that is discoloured from green to brown, shows a reduction of hemoglobin to diffuse methemoglobin. Beta hemolysis (β) is characterized by a transparent, pale-yellow zone encircling the colonies, signifying complete RBC lysis, while gamma hemolysis (ϒ) is characterized by no discernible changes in the medium.

#### MTT cytotoxicity assay

MEM medium containing 10% fetal bovine serum and 1 × antibiotic antimycotic solution was used for plating the Caco-2 cell line into 96-well plates at a density of 1 × 10^4^ cells per well. Subsequent to incubation in a CO2 incubator at 37 °C, 5% CO_2_ and 95% humidity, the culture medium was refreshed on every 48 h and the cells were used 15 days post-appearance of confluence, after complete differentiation. By washing with 200 µl of 1 × PBS, the cells were co-incubated with differential bacterial MOI for 3 h in serum and antibiotic free media. After co-incubation, the cells were washed and resuspended in serum-free medium containing 1 × antibiotic antimycotic solution and 50 µg/ml gentamicin for 1 h, followed by 5 µg/ml gentamicin for 20 h to kill the extracellular bacteria. IC50 of doxorubicin (11 µg/mL) served as a positive control. At the end of the treatment period, the media was aspirated completely from the cells. 0.5 mg/mL MTT (3-[4,5-dimethylthiazol-2-yl]-2,5-diphenyl tetrazolium bromide) solution was added in 1X PBS, and the cells were incubated for 4 h at 37 °C in a CO_2_ incubator. Post-incubation, the media containing MTT was removed, and cells were washed with 200 µl of PBS. After adding 100 µl of DMSO, the formazan crystals were fully dissolved and the colour intensity was measured using microplate reader at 570 nm, as the formazan dye develops blue-purplish colour^22^.

### Confirmation of cell morphology by SEM analysis

On a sterile glass slide, bacterial cells were smeared, allowed to air dry, and then fixed with 4% glutaraldehyde. Following the fixation process, the samples underwent washing in buffer solution (PBS) and dehydrated using ethanol in ascending level concentrations: 20%, 40%, 60%, 80%, and 100%. The sample were carefully dried at 45° overnight. After that, the samples were analysed using a scanning electron microscope operating at 10 kV^23^.

### Molecular identification of the isolate

The most promising isolate exhibiting all positive probiotic characteristic was identified by 16S rRNA gene sequencing utilizing primer- Forward: GGATGAGCCCGCGGCCTA and Reverse: CGGTGTGTACAAGGCCCGG by employing Sanger sequencing. The PCR program was configured using the subsequent cycle parameters: The process involved three minutes of initial denaturation at 94 °C, followed by 30 cycles—denaturation for 1 min at 94 °C, 60 s of annealing—54 °C, and extension for 2 min at 72 °C. The last extension was held for 7 min at 72 °C. The obtained sequence was compared with the GenBank database using the Basic Local Alignment Search Tool (BLAST). This comparison was aimed for the final identification of LAB. Subsequently, the identified sequence was submitted to GenBank and for acquiring of an accession number. A phylogenetic tree was generated using the MEGA 7 program^24,25^.

### Statistical analysis

A detailed data analysis was carried out using GraphPad Prism version 10 software. Comparison between the data was analysed using one way ANOVA. A statistical difference was considered significant at p value less than 0.05 (p < 0.05).

## Results

### Sample collection, isolation, and phenotypic characterisation of endophytic lactic acid bacteria

Initially, six endophytic bacteria isolated from curry leaves were tested for antibacterial efficacy (section "Agar well diffusion assay") against pathogenic microorganisms. The findings revealed that only one isolate (MKL8) showed antibacterial activity compared to the other isolates. The colony morphology was circular, slightly elevated, smooth with entire margin. The isolates were identified as gram-positive under the microscope. They showed a spherical shape with chains (1 µm), were non-motile, and did not generate spores. In terms of biochemical properties, MKL8 was negative for catalase, oxidase, indole, methyl red, citrate, and weakly positive for VP. It was able to ferment carbohydrates (glucose and fructose) while producing acid, but no gas was produced.

### Characteristics of bacteria associated with probiotic activity

#### Resistance to acid and bile salts

**Figure 1:**
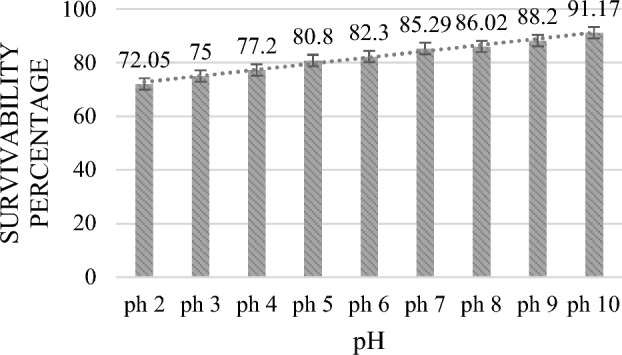
Survival percentage of MKL8 at different pH concentration. Data are expressed as the mean ± standard deviation of three replicates (n = 3); p < 0.05.

**Figure 2:**
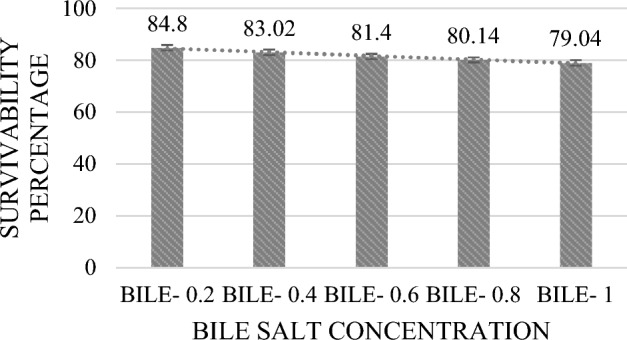
Survival percentage of MKL8 at different bile salt concentration. Data are expressed as the mean ± standard deviation of three replicates (n = 3); p < 0.05.

Potential probiotic strains need to be able to withstand acidic conditions and bile secretions in the stomach and small intestine. Most ingested bacteria die in the gastrointestinal juice because of its pH of 2.0–3.0^26^. In this study, different bile and pH conditions were observed. With a steady increase in pH from 2 to 10 and a survival rate of up to 91.17% (Fig. 1), MKL8 growth increased considerably. Survival rate of 84.8% (Fig. 2) was observed in the case of bile salt tolerance though there was a modest decrease in growth rate with increased bile salt concentration. Therefore, as seen in Figs. 1 and 2, MKL8 demonstrated an impressive pattern of acid and bile tolerance respectively.

#### Resistance to phenol

**Figure 3:**
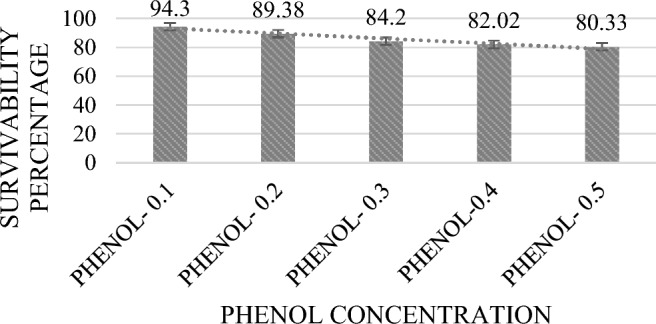
Percentage of survival at varying phenol level. Data are expressed as the mean ± standard deviation of three replicates (n = 3); p < 0.05.

Significant bacterial growth in presence of phenol was observed. Figure 3 illustrates the result of tolerance towards 0.1–0.5% phenol concentration, which was shown by MKL8 after a 24-h incubation period. With a survival rate of more than 80%, the viability of MKL8 differed considerably (P < 0.05) depending on the phenol content.

#### Resistance to NaCl

**Figure 4:**
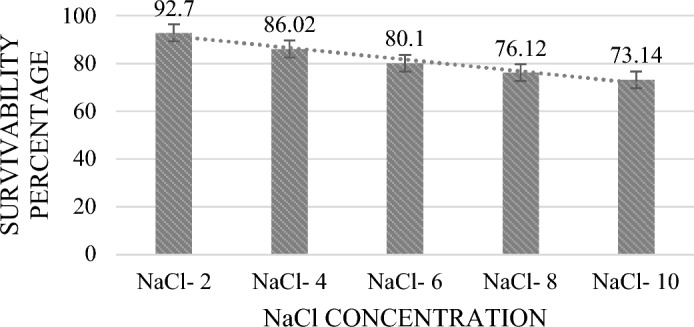
Percentage of survival at different salt concentration of MKL8. Data are expressed as the mean ± standard deviation of three replicates (n = 3); p < 0.05.

MKL8 demonstrated resistance to varying NaCl concentrations between 2 and 10%, however, viability was gradually decreased with an increase in concentration. Nonetheless, after a 24-h incubation period, survival of more than 70% (Fig. 4) was seen. At 2–6% NaCl concentration, the bacteria could proliferate at a steady rate, however, at 8–10%, their growth was gradually reduced. Though it was less than other concentrations, significant growth was still noted at 10.0% NaCl.

#### Resistance to simulated gastric juice

**Figure 5:**
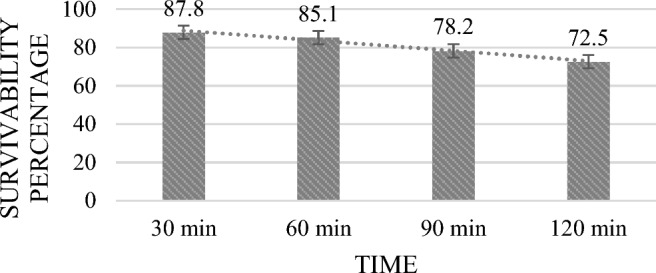
Survival percentage of MKL8 in SGJ. Data are expressed as the mean ± standard deviation of three replicates (n = 3); p < 0.05.

Probiotic bacteria have to withstand in severe GI conditions, including exposure to low pH levels and digestive enzymes like pepsin present in gastric juice. The acidic environment of stomach with a pH ranging from 1.5 to 3.5, it is very crucial for bacteria to survive before selecting possible probiotics. In this work, the survival of isolate in simulated gastric juice at pH 3.0 was studied. Upon being exposed to the simulated environment for 120 min, MKL8 demonstrated a survivable percentage of over 70% (Fig. 5).

### Cell surface and adherence characteristics

#### Auto aggregation assay

**Figure 6:**
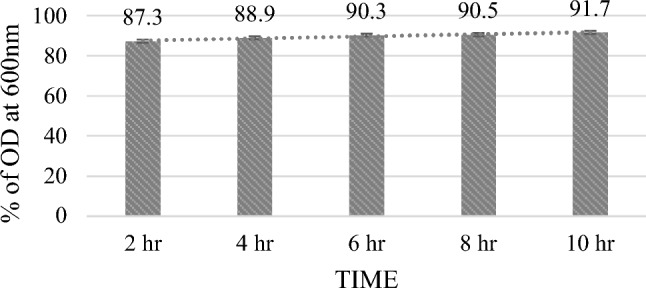
Percentage of auto aggregation of MKL8. Data are expressed as the mean ± standard deviation of three replicates (n = 3); p < 0.05.

LAB often engage in physical interactions with one another and settle down into a static liquid suspension at the bottom by the process of "bacterial auto-aggregation"^27^. MKL8 demonstrated strong adhesion ability at various time intervals, with auto aggregation varying between 87.3 and 91.7% (Fig. 6). The findings revealed that the bacteria could endure in gastrointestinal tract environments and resist stress from the environment.

#### Cell surface hydrophobicity

To determine the isolate's capacity for adhesion, the hydrocarbon n-hexane was used to test for cell surface hydrophobicity. The CSH is a measurement that quantifies the degree of attraction between suspended cells in a water phase and hydrocarbon phase when the two phases are aggressively mixed and allowed to separate. The results of the MKL8's cell surface hydrophobicity analysis was found to be 85.46%.

#### Cell adhesion assay using Caco-2 cells

**Figure 7:**
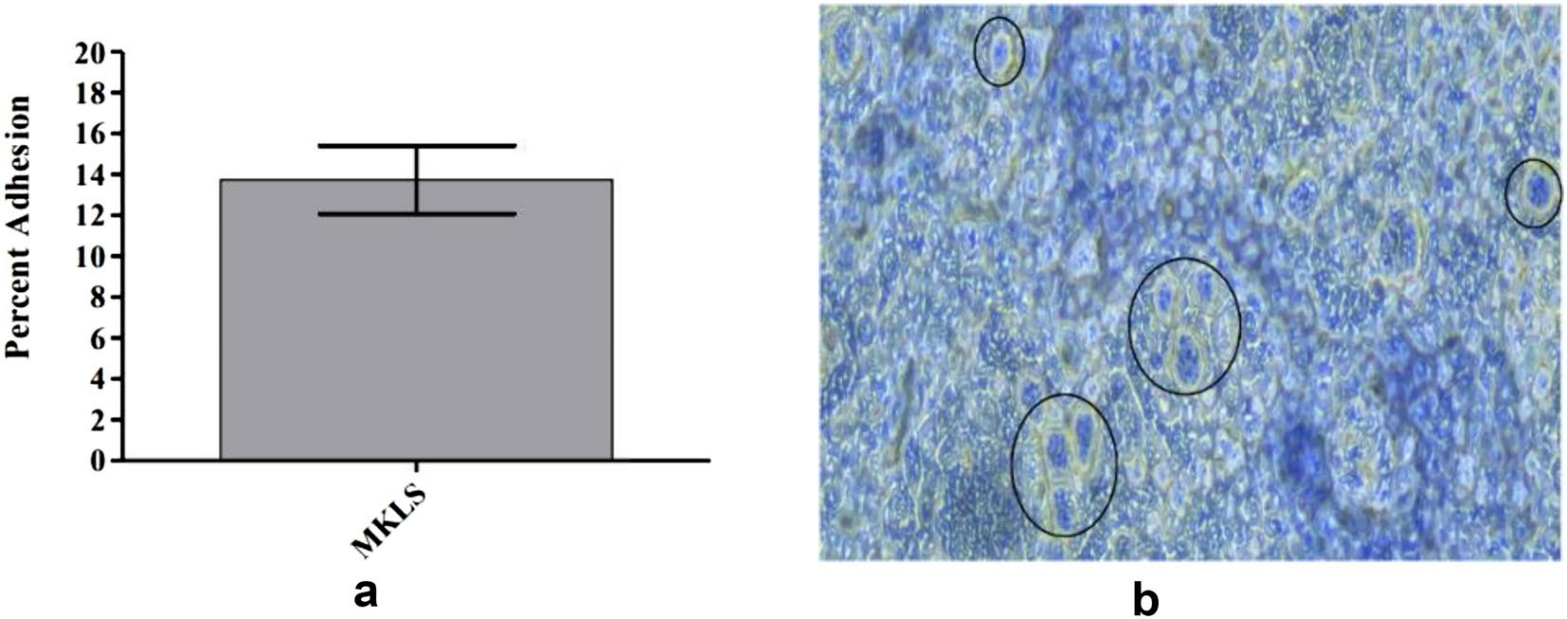
(**a**) Percent adhesion of MKL8. Data are expressed as the mean ± standard deviation of three replicates (n = 3); p < 0.05. (**b**) Microscopic view of MKL8 adhered to Caco-2 cell lines.

Adhesion has consistently been recognized as a optimal measure for assessing the colonization ability of a potential probiotic strain. In addition to enhancing gut immune function to promote the host health, the binding of LAB to the gastrointestinal surface might be imperative for competitively excluding harmful bacteria. Human cell lines such as Caco-2 (non-mucus secreting) have been used in several previous studies to evaluate probiotic strains for adherence. In the current study, the measure of adherence of isolate to Caco-2 was found to be 13.74% (Fig. 7a) and the adhesion abilities of tested strains to Caco-2 cells were observed under OPTIKA inverted microscope (Fig. 7b).

### Antagonistic activity of bacteria

#### Agar well diffusion assay

**Figure 8:**
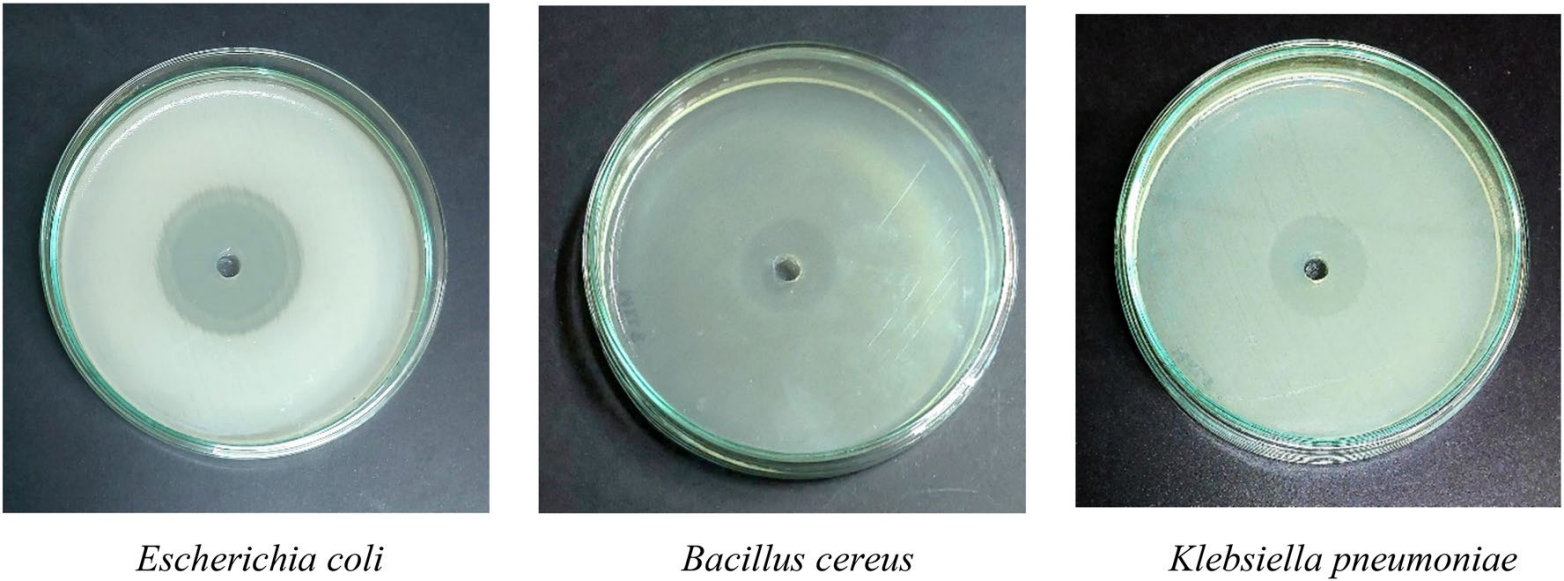
Zone of inhibition against tested pathogens.

MKL8 was used to test its antagonistic activity against three indicator pathogens, which include *Escherichia coli, Klebsiella pneumoniae,* and *Bacillus cereus*. By performing an agar well diffusion test, MKL8 showed varying degrees of inhibitory activity against every pathogen tested (Table 1, Fig. 8). All the tested pathogens having inhibition zones were found to have their development inhibited by the CFS of MKL8.

**Table 1:** Zone of inhibition of MKL8.

	Test pathogen and zone of inhibition		
	*Escherichia coli*	*Bacillus cereus*	*Klebsiella pneumoniae*
MKL8	15 mm	9 mm	11 mm

### Safety evaluation of LAB

#### Hemolytic assay

**Figure 9:**
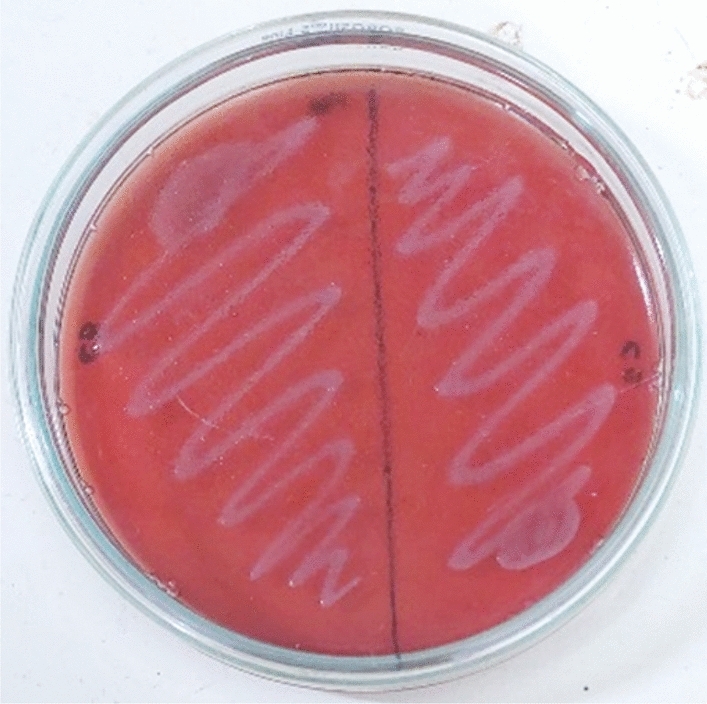
No hemolysis observed in MKL8.

An assessment of MKL8's hemolytic characteristics was conducted to ascertain its pathogenicity. When cultured on blood agar, MKL8 showed γ-hemolytic activity (no hemolysis) and was determined to be negative (Fig. 9).

#### MTT cytotoxicity assay

**Figure 10:**
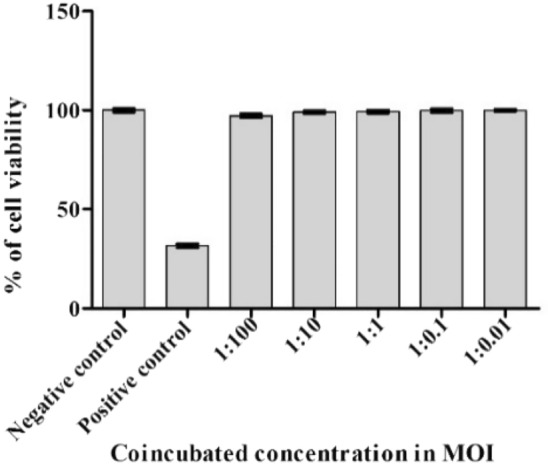
Percent of cell viability of MKL8.

When certain bacterial strains are considered to be used as probiotics, an assessment of their toxicity must be performed. In this work, the impact of MKL8 on Caco-2 cell viability was investigated using the MTT test. Any probiotic strain should not harm cells. Therefore, MKL8 was assessed for its toxicity towards the Caco-2 cells by performing an MTT assay (Fig. 10). The obtained results confirmed that the MKL8 was not toxic to Caco-2 cells and showed cell viability after incubation was found to be 97–100% till 100 MOI. Phase contrast microscopic images at different MOI have been included in the supplementary data S1.

### Confirmation of cell morphology by SEM analysis

**Figure 11:**
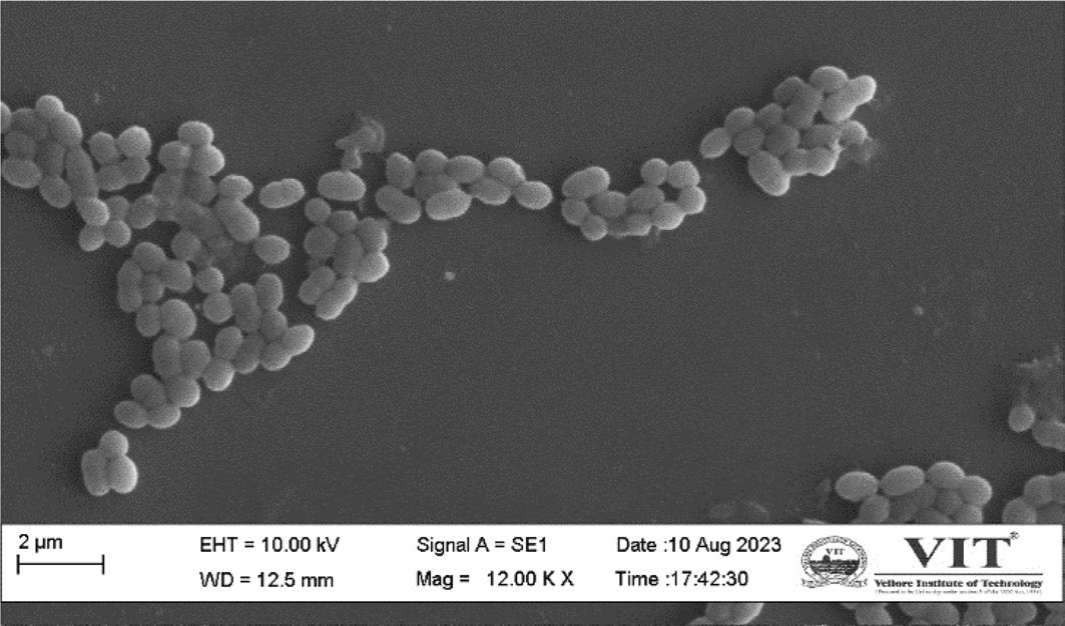
Scanning electron microscopy image of MKL8.

To analyze and confirm the structure and arrangement of MKL8, SEM was used. A 12,000× magnification Scanning Electron Microscopy study was conducted to observe and confirm the structural morphology. The morphological identification of MKL8 was initially performed by gram staining and was observed to be gram-positive and cocci-shaped. To further confirm its morphology, SEM imaging was used as shown in Fig. 11, and the cells appear to have a spherical appearance in pairs and some in the form of chains.

### Molecular identification of the isolate

**Figure 12:**
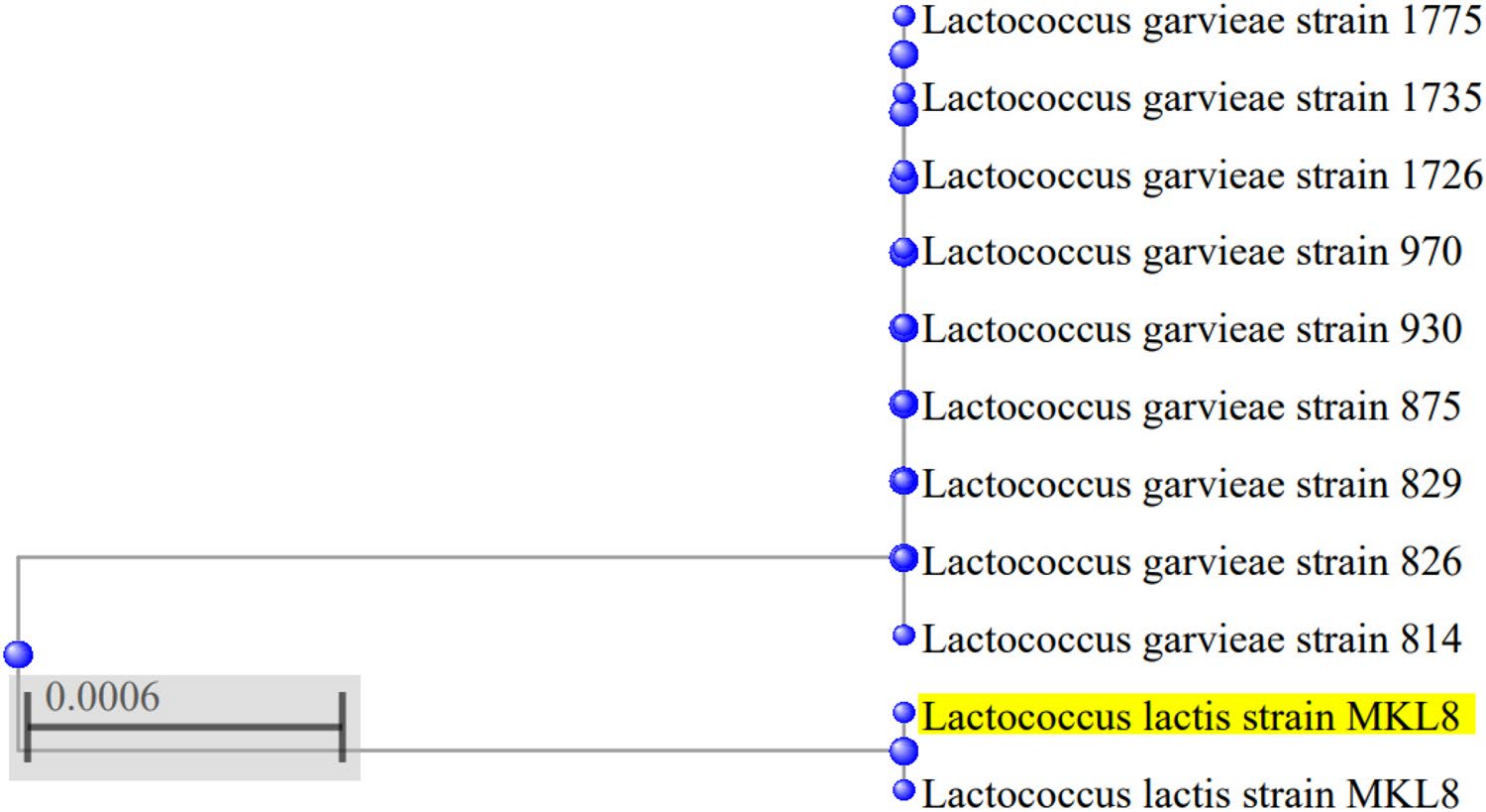
Phylogenetic tree of MKL8.

Using the 16S rRNA sequencing technique, the isolate (MKL8) was identified. The analysis revealed that the selected strain exhibited a 100% sequence similarity. The accession number OR342073 was obtained to *Lactococcus lactis* when the nucleotide sequence was submitted to the GenBank sequence database. Using the neighbor-joining approach, the phylogenetic tree (Fig. 12) for MKL8 was constructed.

## Discussion

Isolation and characterization of probiotic bacteria from unconventional sources, such as plant leaves, remains an understudied area, with the majority of investigations focusing on fermented or dairy food products. In the current study, based on its properties as an effective probiotic, lactic acid bacteria from curry leaf leaves were selected. The bacteria with the best morphological growth characteristics and antibacterial activity was found to be *Lactococcus lactis* (MKL8), and its probiotic and biochemical characteristics were further examined. These findings were based on phenotypic and sequencing analyses. Early research has led to the strong notion that dairy products, such as milk, yogurt^28,29^, cheese and sourdough^30,31^ are the primary source of *Lactococcus sp*. Nonetheless, accumulating evidence from multiple studies has demonstrated the isolation of *Lactococcus* species from non-dairy sources such as fresh vegetables^32^, fruits^33^, leaves^34^, and silage^35^. Moreover, *L. lactis* had also been isolated from the hindguts of termites^36^ and soil^37^. Previous research has also shown that some plant-derived *Lactococcus* sp. have demonstrated technological features, such as: (1) the ability to form flavor-enhancing compounds from amino acids, which are likely advantageous to the dairy sector^38^; (2) producing bacteriocins, which usually inhibit or kill the growth of bacterial strains and have potential uses in pharmaceuticals and food preservation agents^39^; and (3) exhibiting probiotic qualities, such as growth when subjected to 0.3% bile, low acidic conditions, and in vitro cholesterol removal, indicating potential use as probiotic strains^40^. Additionally, *L. lactis* has been employed as a vehicle in delivering therapeutics like cytokines into human body because of it possessing immunomodulatory characteristics. Since *L. lactis* cell wall components have anti-allergic properties through immunomodulatory actions, they are inherently excellent carriers for allergy immunotherapy^41^. Concerning hypersensitivity in regard with food allergies caused by lactose products, *L. lactis* have been used as a treatment as it promotes lactose digestion allowing the consumers to consume without experiencing any discomfort^42^. There have also been several reports that *L. lactis* produces metabolites that is said to have therapeutic use. One such metabolite is non-protein-amino- acid γ-amino butyric acid (GABA), which possess diuretic, hypotensive, anti-cancer, and anti-anxiety characteristics^43^. Along with that, *L. lactis* synthesizes hyaluronic acid which is a carbohydrate-based polymer that finds utility in dermatitis, wound healing, and many cosmetic applications^44^. Given their prospective benefits to human health, research on plant endophytes for isolating LAB is essential. Within plant tissues, endophytes develop special metabolic capabilities that provide them with a wide range of bioactive chemicals with potential medicinal uses. These metabolites have high anti-inflammatory, antioxidant, and antibacterial properties, which in-turn helps with several health issues. Furthermore, endophytes will also be effective and survive in the human gastrointestinal system based on their ability to adapt variety of environmental circumstances within plants. They could be eco-friendly LAB isolating sources because of their flexibility and sustainable source from plant materials. Considering everything, studying plant endophytes for probiotics expands the diversity of microbes for medical applications and creates opportunities for sustainable methods for isolating probiotic bacteria^45^.

The two primary factors identified in hindering the survivability of LAB in the host's GI tract are the presence of bile salt in the duodenum and acid in stomach environment. To ensure that the presumptive LAB would survive in conditions of the gastrointestinal tract, further testing was conducted to determine its tolerance to bile and acid. Consequently, a notable > 80% resistance to bile and acid conditions were observed without any appreciable decrease in viability. The pH of the stomach may reach 3 after consuming food, depending on what is in it. A study by Nami et al. ^46^ analysed the probiotic potential of LAB and their results suggested that *Lactococci* were more tolerant of low-level pH than *Enterococci*. However, Ozdogan et al. studied probiotic and antioxidant qualities of *L. lactis* LL27's and was found that after 4 h at pH 3, the viability dropped by 67.42%. After three hours at pH 2, the vitality of *L. lactis* LL27 was 39.56%; however, after four hours at pH 2, no viable cells were detected. When studied for SGJ, the purpose of the digestion of simulated gastric was to evaluate the ability of LAB isolates to withstand the severe conditions seen in the GI tract. Without experiencing any reduction in viability, the isolate was able to withstand the artificial digestive conditions with over 70% survivability. Research has demonstrated that probiotic bacteria are susceptible to the antimicrobial effects of bile that enter the duodenal region of the small intestine. Bile salts can break down the fatty acid and lipid cell walls found in all bacteria. Thus, it is important to look into whether potential probiotic bacteria can be resilient in the presence of bile salts. Ox-gall contains unconjugated and conjugated bile salts, including glycocholic acid, taurocholic acid, cholesterol, and lecithin. A strain is considered to be an efficient probiotic when it is able to withstand 0.2–0.6% bile salt, which resembles the environment of human intestinal bile salt^47^. In our study, MKL8 demonstrated strong tolerance to all bile salt concentrations, indicating its essential ability to thrive in the gastrointestinal tract (GIT) by resisting bile salts.

With no suppression in viability, MKL8 also showed strong resistance to phenol, ranging from 79 to 94% in all concentrations. According to Xanthopoulos et al.^48^, the ability of LAB to endure 0.6% (w/v) phenol is indicative of its resilience against the potential bacteriostatic effects. This assertion is substantiated by in vitro studies demonstrating that phenol could cause direct damage to the human colonic epithelial cells. Moreover, given that phenols are toxic metabolites resulting from the deamination of specific aromatic amino acids, assessing phenol resistance may offer valuable insights into the survival capabilities of probiotic bacteria under gastrointestinal conditions^49^. MKL8 have also shown tolerance up to 6.0% (w/v) for NaCl, according to the results. Increased NaCl concentrations inhibit the bacteria from growing. This result corresponds with the findings of Mulaw et al.^50^, where they observed that one of the LAB isolates, E052, obtained from traditionally fermented Ethiopian food products, exhibited growth at 4% (w/v) NaCl, but no further growth was observed beyond 6.5% (w/v) NaCl. Because of their tolerance to osmotic conditions, the strains can tolerate the negative effects of high osmotic pressure in the gastrointestinal system while still maintaining a relative osmotic pressure equilibrium. Since bacterial cells present in high salt concentrations will eventually experience a loss of turgor pressure that would alter the enzyme and water activity, physiology, and cell metabolism, the NaCl tolerance test indicates the degree of Osmo tolerance by LAB^51,52^. Tolerance to NaCl and pH is strain- and species-dependent in *Lactococcus* strain, which are homofermentative in nature and contrary to heterofermentative LAB, homofermentative LAB are reported to be more resistant to NaCl^53^.

Probiotic bacteria adhere to and colonize epithelial cells in the gastrointestinal system, thereby preventing pathogen colonization. The crucial function of probiotic bacteria is autoaggregation, which stops the pathogens from colonising the surface. When microorganisms from the same species auto aggregate, they can form self-forming groups. This process is often linked to the bacteria adhering to the intestinal mucosa. These interactions are assessed through cell surface hydrophobicity and auto-aggregation tests^54^. While hydrophobicity is closely related to the ability of strains to adhere to non-polar sources, auto-aggregation is a trait that is beneficial for probiotic adhesion to intestinal cells^55^. In our study, MKL8 demonstrated hydrophobicity of 85% and auto aggregation ranging from 87 to 91%. Similar findings have been reported, with highly aggregating bacteria like *L. salivarius* M2-71 and *L. mucosae* M6-29 showing superior cell adhesion compared to other isolates^56^.

Because probiotics are intended to colonize and attach to the host gut, one of their most crucial characteristics is their ability to adhere to mucosal cells. This adherence is essential for maintaining the viability of LAB in GI tract, thereby enhancing the interactions with host, and aiding in the bacteria's ability to withstand the dynamic conditions of the stomach. Adherence not only prolongs the life of probiotics in the GI tract and improves their interactions with the host, but it also aids in the bacteria's ability to withstand mobility in the stomach. As such, the key characteristic of probiotic bacteria is their capacity in adhere to mucosal surfaces and epithelial cells. This complex adhesion process requires contact between the bacterial cell membrane and the host's mucosal surfaces^57^. Since it is challenging in studying bacterial adherence in-vivo, the human adenocarcinoma cell line Caco-2 is commonly used as an in-vitro model to assess the adhesion capacity of probiotics. Caco-2 cells exhibit the morphological and functional characteristics of normal human intestine cells and hence it makes them suitable for adhesion studies for probiotic bacteria^58^. The adherence level of 30 LAB strains to Caco-2 cells was examined by Morita et al.^59^. The isolates with the highest affinity for Caco-2 cells were *Lactobacillus casei, Bifidobacterium bifidum,* and *Lactobacillus rhamnosus* strain GG. It was revealed that the probiotic bacteria's ability to adhere to surfaces is a strain-dependent feature that is not related to either their origin or taxonomic hierarchy. In our study, the results from the adhesion to CaCo-2 cell experiment were likewise similar to those of autoaggregation and hydrophobicity; high adherence was demonstrated by the contact of components on the cell surface, which is a crucial probiotic trait.

A substantial percentage of foodborne diseases that arise from eating contaminated food are caused by pathogenic microorganisms. Hence, the antibacterial activities of the isolated bacteria were tested in this work using the agar well diffusion method against *Klebsiella pneumoniae, Escherichia coli, Bacillus cereus* which are known to cause a significant number of outbreaks. Against the tested pathogens, the CFS of MKL8 demonstrated significantly strong antibacterial activity. The synthesis of organic acids, with lower pH, hydrogen peroxide, and bacteriocins, is thought to be responsible for the antibacterial action of LAB^60^.

Analysis of hemolytic activity is extremely important as the hemolytic action caused by bacterial hemolysin, causes mild to severe infection by pathogens through lysing red blood cells^61^. Hemolytic activity is generally assessed by observing physical changes resulting from culturing the microbe on a medium containing human or animal blood. In this study, MKL8 exhibited no hemolytic activity on sheep blood agar plates. The trend of our findings aligns with those of Kondrotiene, K., et al.^21^ and Bandyopadhyay et al.^62^. In addition to hemolytic, the potential pathogenicity of LAB is determined by measuring its cytotoxicity on intestinal epithelial cells using a bacterial culture medium. In our study, no toxic effects were associated with MKL8. By using 16S rRNA sequencing and phylogenetic analysis with 100% homology, the isolate MKL8, which showed the highest potential as a probiotic, was confirmed to be *Lactococcus lactis*. Numerous studies are currently exploring the probiotic capabilities of *L. lactis*, which is isolated from a broad range of non-dairy sources, further supporting its application in probiotic research.

## Conclusion

A well-known and widely distributed genus *Lactococcus* sp. may be found naturally in a broad range of foods. The findings of this study indicated that *Lactococcus lactis* (MKL8) isolated from *Murraya koenigii* potentially have high value in industrial food applications as a probiotic. The strain demonstrated favourable properties corresponding to probiotics, including tolerance to bile salts, low pH, phenol, wide range of NaCl, simulated gastric juice and a good inhibitory action. Furthermore, the results of hemolytic activity and MTT studies confirmed its safety, and it demonstrated strong surface binding properties, indicating its ability to survive and colonise the gut in *in-vitro* conditions. Therefore, this study highlights a promising illustration of the biotechnological potential of lactic acid bacteria isolated from *Murraya koenigii*, which allows for more research to investigate other unconventional and novel sources for probiotic bacterial isolation.

### Supplementary Information

Supplementary Information.

## Data Availability

The datasets generated during the current study are available in the GenBank (NCBI-Nucleotide Database) repository under accession number OR342073.
