# Author Correction: Probiotic evaluation, adherence capability and safety assessment of *Lactococcus lactis* strain isolated from an important herb “*Murraya koenigii*”

**DOI:** 10.1038/s41598-024-70191-2

**Published:** 2024-08-26

**Authors:** Tholla Madana Shivani, Mythili Sathiavelu

**Affiliations:** School of Biosciences and Technology, Vellore Institute of Technology, Vellore, 632014 India

Correction to: *Scientific Reports* 10.1038/s41598-024-66597-7, published online 06 July 2024

The original version of this Article contained an error in the name of Tholla Madana Shivani, which was incorrectly given as Shivani Tholla Madana.

In addition, under the Results section, ‘Auto aggregation assay’

“LAB often engage in physical interactions with one another and settle down into a static liquid suspension at the bottom by the process of "bacterial auto-aggregation"^27^. MKL8 demonstrated strong adhesion ability at various time intervals, with auto aggregation varying between 87.3 and 91.7% (Fig. 6). The findings revealed that the bacteria could endure in gastrointestinal tract environments and resist stress from the environment. Figure 7 depicts the visual representation of auto aggregation from the 0th to the 10th hour.”

now reads,

“LAB often engage in physical interactions with one another and settle down into a static liquid suspension at the bottom by the process of "bacterial auto-aggregation"^27^. MKL8 demonstrated strong adhesion ability at various time intervals, with auto aggregation varying between 87.3 and 91.7% (Fig. 6). The findings revealed that the bacteria could endure in gastrointestinal tract environments and resist stress from the environment.

The original Article has been corrected.

