# Supplementary figures and images for: Probiotic evaluation, adherence capability and safety assessment of Lactococcus lactis strain isolated from an important herb “Murraya koenigii”
Source: Sci Rep. 2024 Jul 6;14:15565. doi: 10.1038/s41598-024-66597-7 (PMC11227525; doi:10.1038/s41598-024-66597-7)

**MTT cytotoxicity assay (Fig 11b)**

**
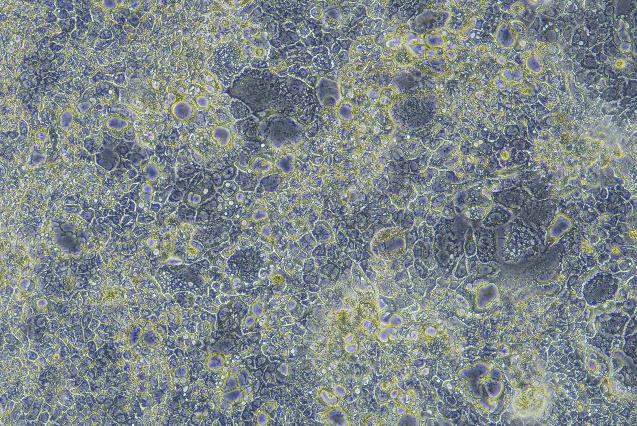

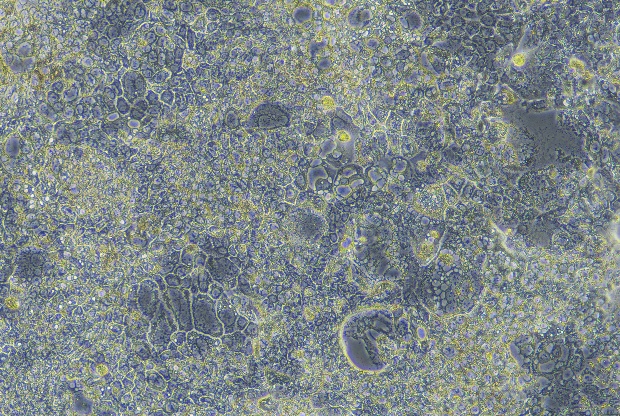
**

**1:0.01 MOI 1:0.1 MOI**

**
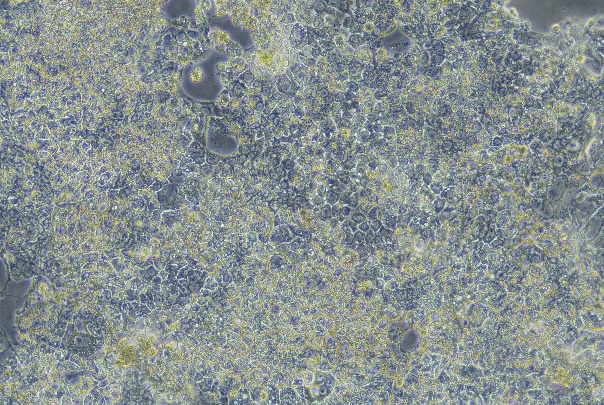

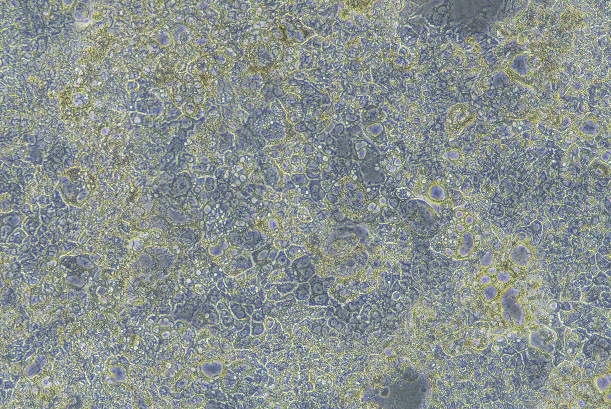
**

**1:1 MOI 1:10 MOI**

**
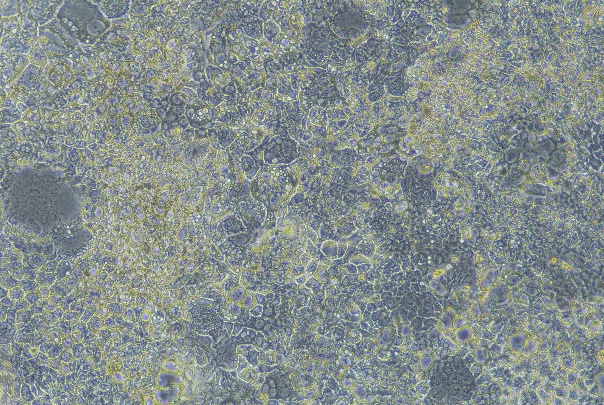
**

**1:100 MOI**

Supplement: Supplementary file 1 — Supplementary Information. [file 41598_2024_66597_MOESM1_ESM.docx]
